# Exploring the causal role of the immune response to varicella-zoster virus on multiple traits: a phenome-wide Mendelian randomization study

**DOI:** 10.1186/s12916-023-02843-5

**Published:** 2023-04-12

**Authors:** Xinzhu Yu, Artitaya Lophatananon, Krisztina Mekli, Kenneth R. Muir, Hui Guo

**Affiliations:** 1 Centre for Biostatistics, Division of Population Health, Health Services Research & Primary Care, School of Health Sciences, Faculty of Biology, Medicine and Health, The University of Manchester, Oxford Road, Manchester, M13 9PL UK; 2 Centre for Integrated Genomic Medicine, Division of Population Health, Health Services Research & Primary Care, School of Health Sciences, Faculty of Biology, Medicine and Health, The University of Manchester, Oxford Road, Manchester, M13 9PL UK

**Keywords:** Varicella- zoster virus, Anti-VZV IgG, Mendelian randomization, MR-PheWAS, MHC

## Abstract

**Background:**

The immune response to infections could be largely driven by the individual’s genes, especially in the major histocompatibility complex (MHC) region. Varicella-zoster virus (VZV) is a highly communicable pathogen. In addition to infection, the reactivations of VZV can be a potential causal factor for multiple traits. Identification of VZV immune response-related health conditions can therefore help elucidate the aetiology of certain diseases.

**Methods:**

A phenome-wide Mendelian randomization (MR) study of anti-VZV immunoglobulin G (IgG) levels with 1370 traits was conducted to explore the potential causal role of VZV-specific immune response on multiple traits using the UK Biobank cohort. For the robustness of the results, we performed MR analyses using five different methods. To investigate the impact of the MHC region on MR results, the analyses were conducted using instrumental variables (IVs) inside (IV_mhc_) and outside (IV_no.mhc_) the MHC region or all together (IV_full_).

**Results:**

Forty-nine single nucleotide polymorphisms (IV_full_) were associated with anti-VZV IgG levels, of which five (IV_mhc_) were located in the MHC region and 44 (IV_no.mhc_) were not. Statistical evidence (false discovery rate < 0.05 in at least three of the five MR methods) for a causal effect of anti-VZV IgG levels was found on 22 traits using IV_mhc_, while no evidence was found when using IV_no.mhc_ or IV_full_. The reactivations of VZV increased the risk of Dupuytren disease, mononeuropathies of the upper limb, sarcoidosis, coeliac disease, teeth problems and earlier onset of allergic rhinitis, which evidence was concordant with the literature. Suggestive causal evidence (*P* < 0.05 in at least three of five MR methods) using IV_full_, IV_mhc_ and IV_no.mhc_ was detected in 92, 194 and 56 traits, respectively. MR results from IV_full_ correlated with those from IV_mhc_ or IV_no.mhc_. However, the results between IV_mhc_ and IV_no.mhc_ were noticeably different, as evidenced by causal associations in opposite directions between anti-VZV IgG and ten traits.

**Conclusions:**

In this exploratory study, anti-VZV IgG was causally associated with multiple traits. IVs in the MHC region might have a substantial impact on MR, and therefore, could be potentially considered in future MR studies.

**Supplementary Information:**

The online version contains supplementary material available at 10.1186/s12916-023-02843-5.

## Background

Varicella-zoster virus (VZV) is a highly communicable pathogen. It is widely present in the general population, with an estimated incidence of 4–4.5 per 1000 person-years [1]. Primary infections of VZV are commonly seen in children as chickenpox. The virus will then remain latent in the sensory dorsal root ganglion cells and can be reactivated throughout life. The reactivations of the virus may cause damage anywhere on the body, from the nerves to the skin [2]. The common manifestations are neurological symptoms, such as radicular pain, itching and unpleasant sensations. The virus can affect the cranial nerve and cause diseases (e.g. tooth exfoliation [3] and facial palsy [2]). Facial palsy could trigger other symptoms such as tinnitus, hearing loss and nausea. VZV reactivation may also result in retinal necrosis and severe ocular diseases [4]. The virus can directly enter the cerebrospinal fluid causing meningitis [5] or invade the spinal cord and produce myelopathy [6]. It is also possible that the virus travels to the arteries to induce vasculopathy like haemorrhagic stroke [7]. Recent studies have shown associations between herpes zoster and other diseases such as depression and anxiety [8]. Therefore, the infection of VZV can have a long-term impact on multiple health conditions.

Humoral immune responses to the same infectious agent vary greatly between individuals. Evidence shows that genetic factors may play a determining role in the individual antibody responses to a variety of viruses, especially the variants in the major histocompatibility complex (MHC) region [9]. Genes likely affect the humoral immune response by controlling the serum immunoglobulin levels, seroconversion rates and intensity of antigen-specific immune responses [10]. Therefore, once a host is exposed to the pathogen, their immune response will be affected by genetic factors. Studying infectious immunity-associated genes helps understand the pathologic mechanisms. Immunoglobulin G (IgG) antibody is the most common antibody in the blood and is persistent throughout a person’s life [11]. It can be used as a stable biomarker of lifetime exposure to viruses. Increased levels of anti-VZV IgG without the presence of herpes zosters implies that it correlates with VZV reactivations [12]. Previous genome-wide association studies (GWASs) investigated single nucleotide polymorphisms (SNPs) that influence the antibody responses against VZV infections. While Petars et.al found no SNPs significantly associated with anti-VZV IgG levels in 1000 healthy individuals [10], five SNPs (rs13197633, rs34073492, rs56401801, rs13204572, rs1048381) were identified in a larger cohort (*N* = 7595) from the UK Biobank [13].

Mendelian randomization (MR) is a well-established tool for inferring causal relationships. By using SNPs as instrumental variables (IVs), MR helps to avoid the issue of unobserved confounding in observational studies. The MHC region is a complex genomic region, in which genes are associated with the risks of many diseases [14]. We hypothesized that IVs in the MHC region may play an important role in MR analysis. However, using MHC IVs in MR has rarely been reported in the literature due to concerns about horizontal pleiotropy [15]. In fact, several robust MR approaches have emerged more recently with the aim to minimize the bias from horizontal pleiotropy and/or outliers. For example, MR-Egger estimation allows a genetic instrument to have a direct effect on the outcome, as long as the direct effect is independent of the instrument’s strength [16]. The weighted median method provides consistent causal estimates if at least 50% of the weights come from valid IVs [17]. MR-RAPS assumes that the pleiotropic effects are balanced and assigns low weights to outliers when estimating the causal effect [18]. The MR-PRESSO method detects the outliers which will then be removed to obtain robust estimations [19]. Nevertheless, each method has its own strengths and weaknesses and requires specific assumptions. It has been recommended that multiple MR methods can be performed to seek concordant evidence for a causal relationship [20].

This study aimed to explore the causal role that VZV-specific immune responses might play in the development of multiple traits, by performing phenome-wide MR studies (MR-PheWAS) between VZV titre measurement and 1370 independent traits using five different MR approaches. We also investigated the impact of the genetic instruments in the MHC region on MR analysis.

## Methods

**Fig. 1:**
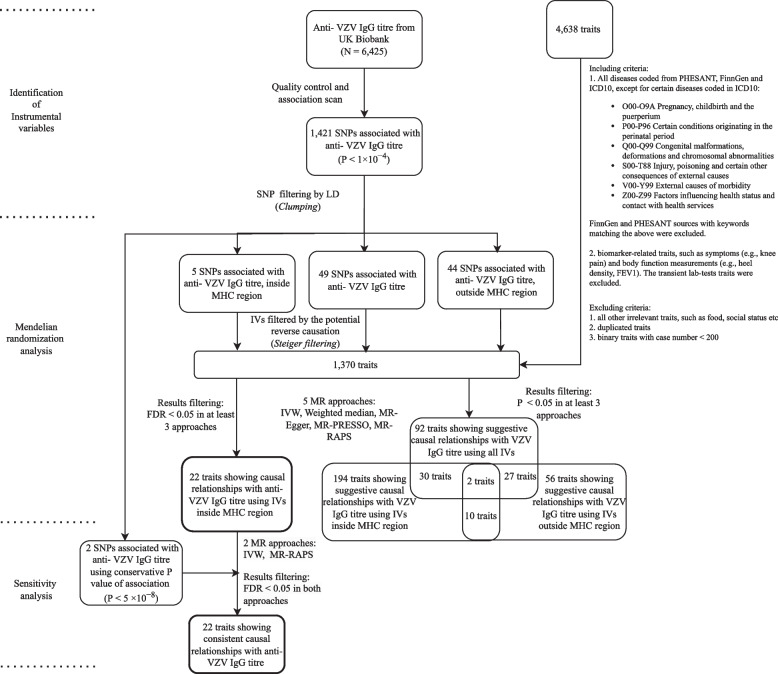
Flowchart of the phenome-wide Mendelian randomization study

The outline of the study is shown in Fig. 1.

### Identification of instrumental variables

The UK Biobank [21] is a large-scale prospective database that has recruited more than 500,000 participants aged between 40 and 69. Within the cohort, 10,000 people were randomly selected to undertake the anti-VZV IgG tests [22]. We extracted genotype data and VZV IgG antibody measurement from the UK Biobank locked in March 2021. After excluding individuals with a high missing rate (missing rate > 0.02), outlying heterozygosity rate (± 3SD from the mean), unmatched sex, non-Caucasians and related individuals, 6425 individuals were included in the analysis. The anti-VZV IgG titres were measured using fluorescent bead-based multiplex serology technology at serum dilution 1:1000 [22]. The output data was median fluorescence intensity values. The titre data was log10-transformed due to skewness. We used linear regression to test for associations between anti-VZV IgG levels and each SNP. SNPs with low minor allele frequency (MAF < 0.01), high missing rate (missing rate > 0.02) and low Hardy–Weinberg equilibrium *P*-value (*P* < 1 × 10^−6^) were removed for downstream analysis. Anti-VZV IgG level-associated SNPs were then carried forward for MR analysis.

Previous MR studies have shown that a conservative *P*-value threshold (5 × 10^−8^) can lead to underpowered MR tests and paradoxical results [23]. In this study, we relaxed the *P-*value threshold to 1 × 10^−4^ to include more IVs because some of the MR methods we used are less prone to weak instrument bias. We then performed the sensitivity analysis using the conventional *P*-value threshold of 5 × 10^−8^. Clumping was carried out on all the neighbouring SNPs (within 10,000 kb) to filter out those in linkage disequilibrium (*R*^2^ ≥ 0.01) and only retained the one with the lowest *P*-value. SNPs selected after clumping were mutually independent and used as IVs for MR analysis. Association analysis between SNPs and anti-VZV IgG, as well as SNP clumping, was conducted using PLINK v1.9.

### Data

We used anti-VZV IgG levels as the exposure. The summary statistics (estimated SNP effects and their standard errors) were extracted from our SNP-anti-VZV IgG association analysis. The Neale Lab GWASs included 361,194 European descendants from the UK Biobank for over 4000 traits, from which we only included diseases and biomarker-related traits as the outcomes. The summary statistics of SNP-outcome associations were obtained from the Neale Lab’s GWASs (round 2, http://www.nealelab.is/uk-biobank). We included traits coded from PHESANT, FinnGen and ICD10. Certain diseases such as injuries and chromosomal abnormalities were excluded (details of the criteria code are in Additional file 1: Text S1). We also included the biomarker-related traits, such as symptoms (e.g. knee pain) and body function measurements (e.g. heel density, forced expiratory volume in 1-s (FEV1)). The transient function measurements (e.g. microalbumin in urine, blood cell count, creatinine (quantile)) which fluctuate over time were removed. For continuous traits, we selected rank-normalized data. For binary traits, we filtered out those with a small number of cases (< 200) to increase the statistical power. For those traits measured in multiple ways, we only reported one of them following the criteria of (1) doctor-diagnosed rather than self-reported, (2) the one reflecting general condition rather than period-specific (e.g. general alcohol intake frequency vs alcohol consumption in last few days), and (3) for duplicated traits with the same phenotype descriptions, the one with a larger number of cases, less missingness or newer versions. We used single-gender data for gender-related diseases (e.g. female-only GWAS results for ovary-related traits). We used GWAS results of both sexes for all the remaining traits.

### MR analysis

To investigate the impact of the MHC region on MR results, we partitioned the IVs by MHC region: in (IV_mhc_) and outside the region (IV_no.mhc_). For each trait, we performed three sets of MR tests—MHC (IV_mhc_), non-MHC (IV_no.mhc_) and combined IVs (IV_full_). The MHC region was defined as chr6:28,477,797–33,448,354 (GRCh37) (the Genome Reference Consortium [24]). Harmonization was conducted for IVs between exposure and outcome datasets to ensure the same minor allele was used for each IV. Steiger filtering [25] was applied such that SNPs showing a stronger association with the outcome than with the exposure (*P*_Steiger_ < 0.05) were excluded. MR analyses were conducted using five methods: random effect inverse-variance weighted (IVW) estimation [20] and four robust methods including weighted median [17], MR-Egger [16], MR-PRESSO [19] and MR-RAPS [18]. In the IVW analysis, the second-order term from the delta expansion was selected for the variance expression to better capture the uncertainty in the estimated causal effect from each SNP. Cochran’s *Q* tests were examined for heterogeneity across all instruments. The MR-Egger intercept test was performed to examine the direct pleiotropic effect of IVs on outcomes. The MR-PRESSO global and distortion tests were conducted to detect the effect of outliers on the results and adjust the estimated causal effects accordingly. The Benjamini-Hochberg’s false discovery rate (FDR) correction was applied to account for multiple testing [26]. We regarded the relationship between anti-VZV IgG levels and a trait as suggestive if there was statistical evidence (*P* < 0.05) in at least three out of the five methods. It was considered causal evidence if FDR < 0.05 in at least three MR methods. Sensitivity analyses were performed using *P* < 5 × 10^−8^ for IV filtering, where only two SNPs remained for IVW and MR-RAPS analyses as the other methods were not applicable. If FDR < 0.05 in both methods, it was regarded as consistent causal evidence. For those traits showing suggestive causal relationships with the anti-VZV IgG levels, *Z*-statistic from each MR analysis was used to compare the results from different methods and IVs. The Spearman correlation test was conducted to evaluate the correlations between the MR estimates from different sets of IVs. MR analyses were implemented using ‘MRPRESSO’, ‘TwoSampleMR’ and ‘MendelianRandomization’ packages in R. The R code for the analysis can be found in Additional file 1: Code S1.

## Results

The mean anti-VZV IgG levels (log10-transformed) and standard deviation were 2.724 and 0.514, ranging from 0 to 4.021. As many as 1421 SNPs were found associated with anti-VZV IgG, of which 1205 (84.8%) were in the MHC region. The complete list of these SNPs was provided in Additional file 2: Table S1. Four of the SNPs (rs13197633, rs13204572, rs1048381 and rs56401801) were also associated with anti-VZV IgG in a previous UK biobank GWAS [13]. After clumping, 49 independent anti-VZV IgG-associated SNPs with F-statistic greater than 10 were selected as IVs (IV_full_) for MR analysis, among which 5 SNPs were inside (IV_mhc_) and 44 were outside the MHC region (IV_no.mhc_) (Table 1). The average *F*-statistic for IV_mhc_, IV_no.mhc_ and IV_full_ was 40, 19.1 and 16.7, respectively. The numbers of the IVs used in MR tests and the SNPs removed after Steiger filtering were listed in Additional file 2: Table S2. A total of 1370 traits (73 ordinal, 773 categorical, 459 binary and 65 continuous) were included in the MR analysis. Additional file 2: Table S2 listed all these results.

**Table 1:** Instrumental variables identified from the genome-wide association scan

SNP	CHR	Position	Minor allele	Beta	SE	MAF	*P* value	*F*-statistic	*R*^2^	MHC region
rs749003	1	118,531,980	C	0.053	0.013	0.142	4.98E − 05	16.479	0.003	No
rs843971	1	153,277,423	T	− 0.037	0.009	0.367	7.71E − 05	15.647	0.002	No
rs114925113	1	170,714,714	A	− 0.139	0.033	0.017	2.63E − 05	17.679	0.003	No
rs2642449	1	220,985,677	C	− 0.060	0.013	0.139	3.06E − 06	21.819	0.003	No
rs708108	1	228,189,855	T	− 0.037	0.009	0.402	8.41E − 05	15.482	0.002	No
rs74482711	2	127,357,322	G	− 0.067	0.016	0.092	3.74E − 05	17.013	0.003	No
rs34499171	2	129,591,736	T	0.045	0.011	0.192	7.62E − 05	15.667	0.002	No
rs61731470	2	211,085,491	A	0.112	0.028	0.028	5.96E − 05	16.132	0.003	No
rs12471761	2	241,310,093	G	− 0.110	0.028	0.028	7.47E − 05	15.702	0.002	No
rs17495909	3	1,323,130	C	− 0.097	0.022	0.042	1.22E − 05	19.169	0.003	No
rs809764	3	61,958,023	G	− 0.039	0.010	0.327	5.27E − 05	16.372	0.003	No
rs116664692	3	62,271,345	G	0.180	0.046	0.010	8.90E − 05	15.380	0.002	No
rs11706859	3	100,713,091	C	− 0.137	0.034	0.019	7.03E − 05	15.819	0.002	No
rs35573656	3	126,612,552	C	− 0.073	0.018	0.072	3.00E − 05	17.442	0.003	No
rs73112967	4	27,778,474	C	− 0.083	0.020	0.055	5.44E − 05	16.302	0.003	No
rs156572	4	155,739,138	T	− 0.109	0.025	0.036	1.23E − 05	19.141	0.003	No
rs72696744	4	163,745,634	T	0.117	0.029	0.025	4.75E − 05	16.577	0.003	No
rs1395479	4	178,318,191	A	− 0.043	0.010	0.264	2.68E − 05	17.650	0.003	No
rs78827810	4	186,683,778	G	− 0.115	0.029	0.026	8.06E − 05	15.553	0.002	No
rs56244403	6	24,017,867	T	− 0.038	0.010	0.383	8.13E − 05	15.546	0.002	No
rs12193110	6	29,937,104	T	− 0.065	0.010	0.299	8.83E − 11	42.179	0.007	Yes
rs17200698	6	31,483,700	T	− 0.087	0.022	0.049	5.14E − 05	16.425	0.003	Yes
rs116462901	6	31,488,532	C	− 0.138	0.032	0.020	2.17E − 05	18.059	0.003	Yes
rs3129888	6	32,411,726	G	− 0.058	0.011	0.194	2.66E − 07	26.544	0.004	Yes
rs1048381	6	32,610,445	A	0.116	0.012	0.171	9.28E − 23	97.288	0.015	Yes
rs80018618	6	37,337,516	A	0.161	0.039	0.013	4.04E − 05	16.864	0.003	No
rs41265357	6	71,238,178	G	− 0.186	0.047	0.010	7.06E − 05	15.816	0.002	No
rs2180346	6	168,944,598	C	0.072	0.015	0.106	9.25E − 07	24.110	0.004	No
rs72695580	9	6,632,950	G	0.170	0.042	0.014	6.02E − 05	16.118	0.003	No
rs11139344	9	84,239,037	C	0.042	0.010	0.268	3.49E − 05	17.164	0.003	No
rs116919233	9	119,991,655	T	− 0.123	0.031	0.021	8.77E − 05	15.398	0.002	No
rs1072523	10	28,819,406	A	0.036	0.009	0.492	8.63E − 05	15.436	0.002	No
rs3759012	11	118,901,262	T	0.036	0.009	0.386	8.99E − 05	15.356	0.002	No
rs11609865	12	105,952,417	C	− 0.073	0.018	0.068	5.83E − 05	16.173	0.003	No
rs116950913	12	132,889,076	A	− 0.101	0.025	0.038	4.66E − 05	16.605	0.003	No
rs75782087	13	38,822,878	T	− 0.096	0.023	0.037	3.94E − 05	16.932	0.003	No
rs111259346	13	39,377,730	G	− 0.092	0.024	0.034	9.86E − 05	15.181	0.002	No
rs73241676	13	82,454,247	G	0.055	0.014	0.131	5.36E − 05	16.332	0.003	No
rs72740509	15	56,533,519	T	− 0.055	0.014	0.123	6.59E − 05	15.954	0.002	No
rs9747501	17	2,739,061	A	0.043	0.010	0.260	3.62E − 05	17.085	0.003	No
rs12948236	17	65,633,567	C	0.037	0.009	0.426	6.97E − 05	15.841	0.002	No
rs6507201	18	35,016,217	A	0.038	0.009	0.359	6.26E − 05	16.042	0.002	No
rs319445	18	55,360,524	A	0.055	0.014	0.122	8.86E − 05	15.390	0.002	No
rs622989	19	7,574,308	C	0.127	0.032	0.020	6.79E − 05	15.875	0.002	No
rs34335847	19	14,705,499	A	− 0.061	0.015	0.102	4.25E − 05	16.780	0.003	No
rs45465702	20	33,320,571	C	− 0.050	0.012	0.184	2.04E − 05	18.189	0.003	No
rs78492969	20	49,248,213	T	− 0.101	0.024	0.037	2.39E − 05	17.868	0.003	No
rs62222450	21	40,471,448	T	0.073	0.018	0.063	6.07E − 05	16.110	0.003	No
rs117884976	22	28,107,887	T	− 0.112	0.029	0.024	9.00E − 05	15.359	0.002	No

*F-statistic*, *F*-statistic of the SNP-exposure association test, *R*^*2*^, the proportion of variance in the anti-VZV IgG level explained by a given SNP, *MAF* minor allele frequency

### MR results using IVs in the MHC region (IV_mhc_)

Suggestive causal relationships (*P* < 0.05 in at least three of the five MR methods) were shown between anti-VZV IgG and 194 traits, among which the results of 86, 104 and 4 traits passed the nominal *P* value threshold 0.05 in three, four and five MR methods, respectively. Elevated anti-VZV IgG levels increased the risks of cellulitis, disease of the blood and blood-forming organs and certain disorders involving the immune mechanism, mouth/teeth dental problems: dentures and other serious medical condition/disability diagnosed by a doctor (*P* < 0.05 in all five MR methods).

**Fig. 2:**
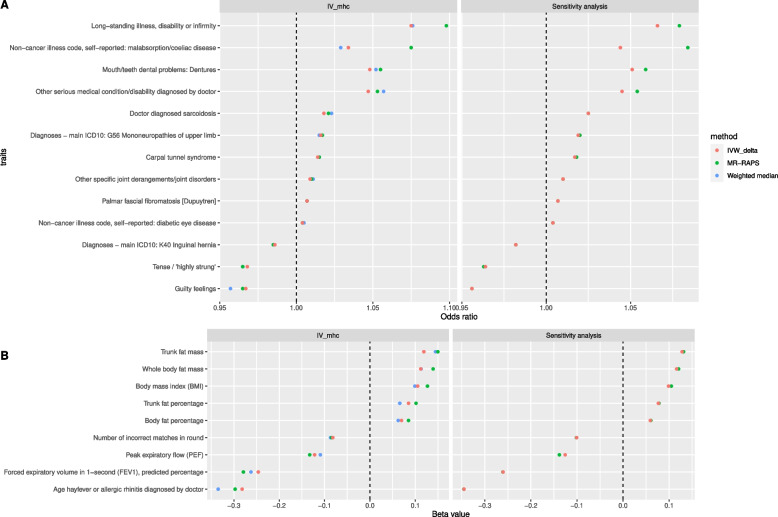
Traits causally affected by anti-VZV IgG levels. Listed are traits with statistical evidence (*FDR* < 0.05) in at least three Mendelian randomization (MR) methods, using instrumental variables inside the MHC region (IV_mhc_) (left panel). The results are consistent in the sensitivity analyses (right panel). Odds ratio (**A**, horizontal axis), for a binary case/control trait, represents the estimated odds ratio when the anti-VZV IgG levels increase by 9 times. Beta value (**B**, horizontal axis), for a continuous trait, represents the estimated change in the tested trait when the anti-VZV IgG levels increase by 9 times

Causal evidence (FDR < 5% in at least three of the five MR methods) was found between anti-VZV IgG and 22 traits, which appeared to be consistent causal evidence because all the results had FDR < 5% in both the MR methods in the sensitivity analysis (Table 2, Fig. 2). Increased level of anti-VZV IgG led to a higher risk of 10 health conditions, including diabetic eye disease, palmar fascial fibromatosis/Dupuytren disease, other specific joint derangements/joint disorders, carpal tunnel syndrome, mononeuropathies of the upper limb, sarcoidosis, malabsorption/coeliac disease, other serious medical conditions/disabilities, mouth/teeth dental problems: dentures and long-standing illness, disability, or infirmity. Anti-VZV IgG also showed a positive causal effect on body fat mass and percentage, trunk fat mass and percentage and body mass index (BMI), which suggested that VZV reactivations increased the chance of being obese.

**Table 2:** MR results of traits causally affected by anti-VZV IgG levels

Phenotype description	Sample size	Case	Control	Source	IV	IVW		MR-Egger		MR-PRESSO		MR-RAPS		Weighted median	
						Beta	FDR	Beta	FDR	Beta	FDR	Beta	FDR	Beta	FDR
Age hay fever or allergic rhinitis diagnosed by a doctor	20,904	NA	NA	phesant	IV_mhc_	− 0.282	0.045	− 0.461	0.979	− 0.29	0.329	− 0.297	0.002	− 0.335	0.01
					Sensitivity analysis	− 0.345	0.004	NA	NA	NA	NA	− 0.346	0.004	NA	NA
Body fat percentage	354,628	NA	NA	phesant	IV_mhc_	0.07	0.043	0.023	0.994	0.08	0.359	0.085	0	0.062	0.017
					Sensitivity analysis	0.059	0.011	NA	NA	NA	NA	0.06	0.01	NA	NA
Body mass index (BMI)	359,983	NA	NA	phesant	IV_mhc_	0.105	0.027	0.197	0.979	0.119	0.332	0.127	0	0.099	0.002
					Sensitivity analysis	0.099	0.001	NA	NA	NA	NA	0.105	0	NA	NA
Forced expiratory volume in 1-s (FEV1), predicted percentage	117,241	NA	NA	phesant	IV_mhc_	− 0.246	0.019	− 0.197	0.979	− 0.264	0.326	− 0.279	0	− 0.262	0
					Sensitivity analysis	− 0.26	0	NA	NA	NA	NA	− 0.261	0	NA	NA
Number of incorrect matches in round	360,686	NA	NA	phesant	IV_mhc_	− 0.082	0.01	− 0.057	0.979	− 0.084	0.326	− 0.085	0	− 0.087	0.002
					Sensitivity analysis	− 0.101	0	NA	NA	NA	NA	− 0.101	0	NA	NA
Peak expiratory flow (PEF)	329,404	NA	NA	phesant	IV_mhc_	− 0.122	0	− 0.06	0.979	− 0.129	0.301	− 0.133	0	− 0.109	0
					Sensitivity analysis	− 0.126	0	NA	NA	NA	NA	− 0.138	0	NA	NA
Trunk fat mass	354,597	NA	NA	phesant	IV_mhc_	0.119	0.043	0.27	0.979	0.132	0.329	0.15	0	0.144	0
					Sensitivity analysis	0.128	0	NA	NA	NA	NA	0.131	0	NA	NA
Trunk fat percentage	354,619	NA	NA	phesant	IV_mhc_	0.085	0.019	0.053	0.979	0.096	0.335	0.102	0	0.066	0.031
					Sensitivity analysis	0.077	0.005	NA	NA	NA	NA	0.078	0.004	NA	NA
Whole body fat mass	354,244	NA	NA	phesant	IV_mhc_	0.112	0.043	0.257	0.979	0.108	0.329	0.139	0	0.113	0.001
					Sensitivity analysis	0.116	0	NA	NA	NA	NA	0.121	0	NA	NA
						Odds ratio		Odds ratio		Odds ratio		Odds ratio		Odds ratio	
Carpal tunnel syndrome	361,194	7973	353,221	finngen	IV_mhc_	1.014	0.045	1.022	0.979	1.014	0.329	1.015	0	1.015	0.007
					Sensitivity analysis	1.017	0	NA		NA		1.018	0	NA	
Diagnoses—main ICD10: G56 mononeuropathies of upper limb	361,194	8130	353,064	icd10	IV_mhc_	1.016	0.011	1.026	0.979	1.016	0.326	1.017	0	1.015	0.008
					Sensitivity analysis	1.019	0	NA		NA		1.02	0	NA	
Diagnoses—main ICD10: K40 Inguinal hernia	361,194	13,147	348,047	icd10	IV_mhc_	0.986	0.047	0.965	0.979	0.985	0.329	0.985	0	0.985	0.025
					Sensitivity analysis	0.982	0.001	NA		NA		0.982	0.001	NA	
Doctor diagnosed sarcoidosis	91,787	395	91,392	phesant	IV_mhc_	1.018	0.043	1.04	0.979	1.02	0.328	1.021	0	1.023	0
					Sensitivity analysis	1.025	0	NA		NA		1.025	0	NA	
Guilty feelings	351,907	100,128	251,779	phesant	IV_mhc_	0.967	0.045	0.966	0.979	0.966	0.329	0.965	0.001	0.957	0.002
					Sensitivity analysis	0.956	0.001	NA		NA		0.956	0.001	NA	
Long-standing illness, disability or infirmity	352,798	114,798	238,000	phesant	IV_mhc_	1.075	0.043	1.099	0.979	1.094	0.326	1.098	0	1.076	0
					Sensitivity analysis	1.066	0	NA		NA		1.079	0	NA	
Mouth/teeth dental problems: dentures	359,841	60,977	298,864	phesant	IV_mhc_	1.048	0.01	1.118	0.979	1.052	0.326	1.055	0	1.052	0.001
					Sensitivity analysis	1.051	0	NA		NA		1.059	0	NA	
Non-cancer illness code, self-reported: diabetic eye disease	361,141	699	360,442	phesant	IV_mhc_	1.004	0.001	1.006	0.979	1.004	0.301	1.004	0	1.005	0
					Sensitivity analysis	1.004	0.001	NA		NA		1.004	0.001	NA	
Non-cancer illness code, self-reported: malabsorption/coeliac disease	361,141	1587	359,554	phesant	IV_mhc_	1.034	0.045	1.12	0.979	1.036	0.484	1.075	0	1.029	0
					Sensitivity analysis	1.044	0	NA		NA		1.084	0	NA	
Other serious medical condition/disability diagnosed by a doctor	354,588	72,965	281,623	phesant	IV_mhc_	1.047	0.043	1.119	0.979	1.051	0.329	1.053	0	1.057	0
					Sensitivity analysis	1.045	0	NA		NA		1.054	0	NA	
Other specific joint derangements/joint disorders	361,194	7943	353,251	finngen	IV_mhc_	1.009	0.05	1.008	0.979	1.009	0.326	1.01	0.011	1.011	0.022
					Sensitivity analysis	1.01	0.042	NA		NA		1.01	0.04	NA	
Palmar fascial fibromatosis [Dupuytren]	361,194	2948	358,246	finngen	IV_mhc_	1.007	0.01	1.006	0.979	1.007	0.325	1.007	0	1.007	0.027
					Sensitivity analysis	1.007	0.02	NA		NA		1.007	0.022	NA	
Tense/ ‘highly strung’	350,159	60,513	289,646	phesant	IV_mhc_	0.968	0.043	0.956	0.979	0.966	0.329	0.965	0	0.965	0.005
					Sensitivity analysis	0.964	0.001	NA		NA		0.963	0.001	NA	

*FDR* Benjamini-Hochberg’s false discovery rate

*NA* not applicable

*IVW* inverse-variance weighted method, *IVmhc* instrumental variables using SNPs in the MHC region

Negative causal associations with anti-VZV IgG levels were found in seven traits. A higher level of anti-VZV IgG resulted in a diagnosis of hay fever or allergic rhinitis at a younger age and worse performance in the expiratory test including predicted FEV1 percentage and peak expiratory flow (PEF), meaning that VZV reactivation might have a detrimental effect on respiratory functions. However, anti-VZV IgG appeared to be a protective factor of four traits because those with higher anti-VZV IgG had a smaller number of incorrect matches in intelligence tests and a lower chance of having hernia, guilty feelings and tension.

### MR results using IVs not in the MHC region (IV_no.mhc_)

By using IV_no.mhc_, we found suggestive causal evidence between anti-VZV IgG levels and 56 traits, among which the results of 42, 12 and 2 traits passed the nominal *P* value threshold in three, four and five MR methods, respectively. The suggestive causal associations of two traits (body fat percentage and mouth/teeth dental problems: dentures) with anti-VZV IgG were also found when using IV_mhc_ (Table 2) and with the estimated causal effects on the contrary direction. None of these suggestive causal signals remained after FDR correction.

### MR results using IVs from all regions (IV_full_)

Suggestive evidence for a causal relationship with anti-VZV IgG levels was found in 92 traits, among which 33 passed the nominal *P* value threshold in 4 MR methods and 9 traits in all five methods. Three of the traits (other specific joint derangements/joint disorders, malabsorption/coeliac disease and tension) showing suggestive causal associations with anti-VZV IgG also showed the same evidence when using IV_mhc_ (Table 2) and with the estimated causal effects in the same directions. Again, no causal evidence passed FDR correction.

### Comparisons of MR results using different sets of IVs (IV_mhc_, IV_no.mhc_, IV_full_)

**Fig. 3:**
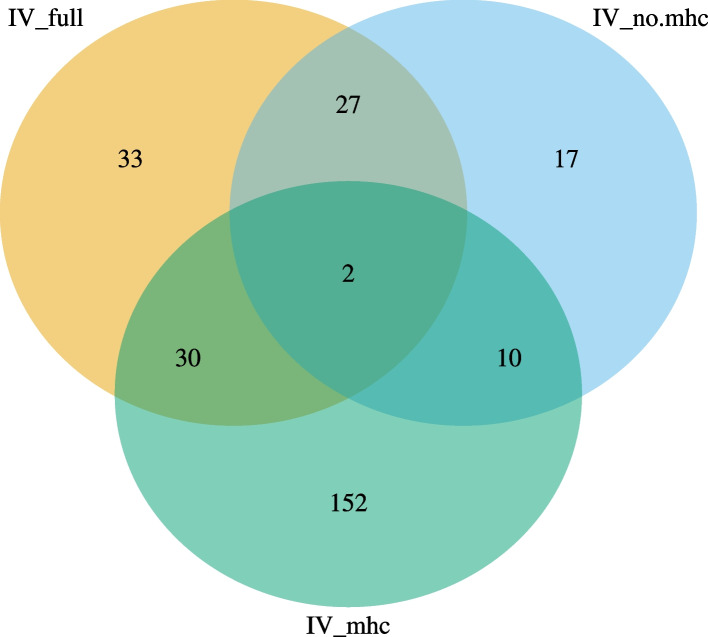
Venn diagram of suggestive causal results using IVs inside, outside MHC region or all IVs. The Venn diagram showed the number of traits that were suggestively casually associated (*P* < 0.05 in at least three Mendelian randomization methods) with anti-VZV IgG levels using instrumental variables inside MHC region (IV_mhc_) (green circle), outside MHC region (IV_no.mhc_) (blue circle) and combined IVs (IV_full_) (yellow circle). Numbers in the overlapping region represent the causal traits identified by both or all IV sets

**Fig. 4:**
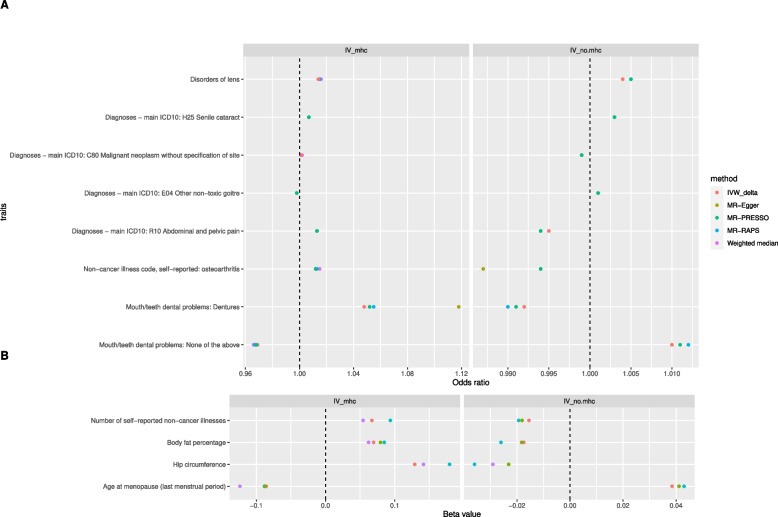
Traits suggestively causally affected by anti-VZV IgG levels using IVs inside or outside the MHC region. Listed are traits with suggestive statistical evidence (*P* < 0.05) in at least three Mendelian randomization (MR) methods, using instrumental variables inside the MHC region (IV_mhc_) (left panel) or outside the MHC region (IV_no.mhc_) (right panel). Odds ratio (**A**, horizontal axis), for a binary case/control trait, represents the estimated odds ratio when the anti-VZV IgG levels increase by 9 times. Beta value (**B**, horizontal axis), for a continuous trait, represents the estimated change in the tested trait when the anti-VZV IgG levels increase by 9 times. Except for disorders of lens and senile cataract, the causal estimates of other traits with the exposure are in the opposite directions between IV_mhc_ and IV_no.mhc_

**Fig. 5:**
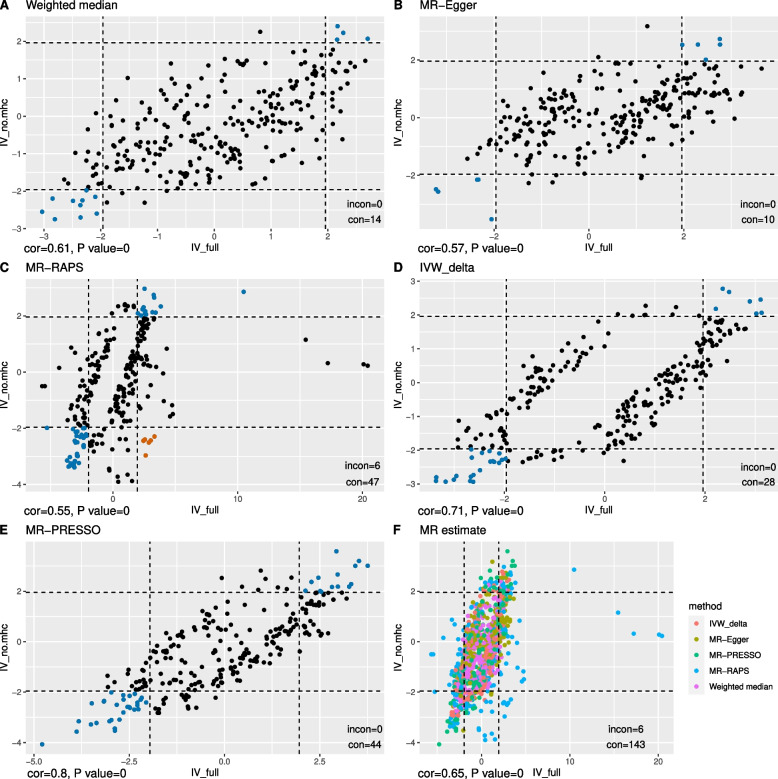
Comparisons between MR estimates inferred from all IVs and IVs outside the MHC region. Scatter plot of the *Z* statistics of MR estimates of traits showing suggestive causal evidence with anti-VZV IgG levels using IVs outside MHC region (IV_no.mhc_, vertical axis) or all IVs (IV_full_, horizontal axis). **A**–**E** Results inferred from one MR method, with blue and orange dots indicating consistent (in the same causal directions) or inconsistent (in the opposite causal directions) MR results separately. **F** The combined MR results inferred from different methods, which are suggested by different colours. The dashed lines are the 95% confidence interval (CI) of *Z* statistics. Dots outside the CI are statistically significant (*P* < 0.05) MR results. The annotation on the bottom-left of each figure shows the Spearman correlation estimate (cor) and *P*-value of the correlation test. The legend on the bottom-right of each figure represents the number of the results that are statistically consistent (con) and statistically inconsistent (incon)

**Fig. 6:**
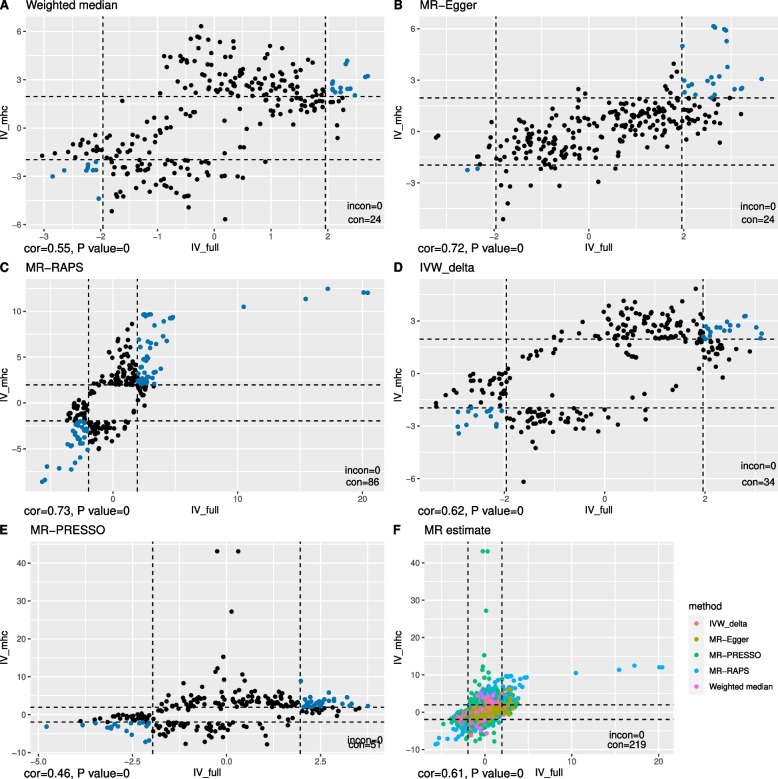
Comparisons between MR estimates inferred from all IVs and IVs inside the MHC region. Scatter plot of the *Z* statistics of MR estimates of traits showing suggestive causal evidence with anti-VZV IgG levels using IVs outside MHC region (IV_mhc_, vertical axis) or all IVs (IV_full_, horizontal axis). **A**–**E** The results inferred from one MR method, with blue and orange dots indicating consistent (in the same causal directions) or inconsistent (in the opposite causal directions) MR results separately. **F** The combined MR results inferred from different methods, which are suggested by different colours. The dashed lines are the 95% confidence interval (CI) of *Z* statistics. Dots outside the CI are statistically significant (*P* < 0.05) MR results. The annotation on the bottom-left of each figure shows the Spearman correlation estimate (cor) and *P*-value of the correlation test. The legend on the bottom-right of each figure represents the number of the results that are statistically consistent (con) and statistically inconsistent (incon)

**Fig. 7:**
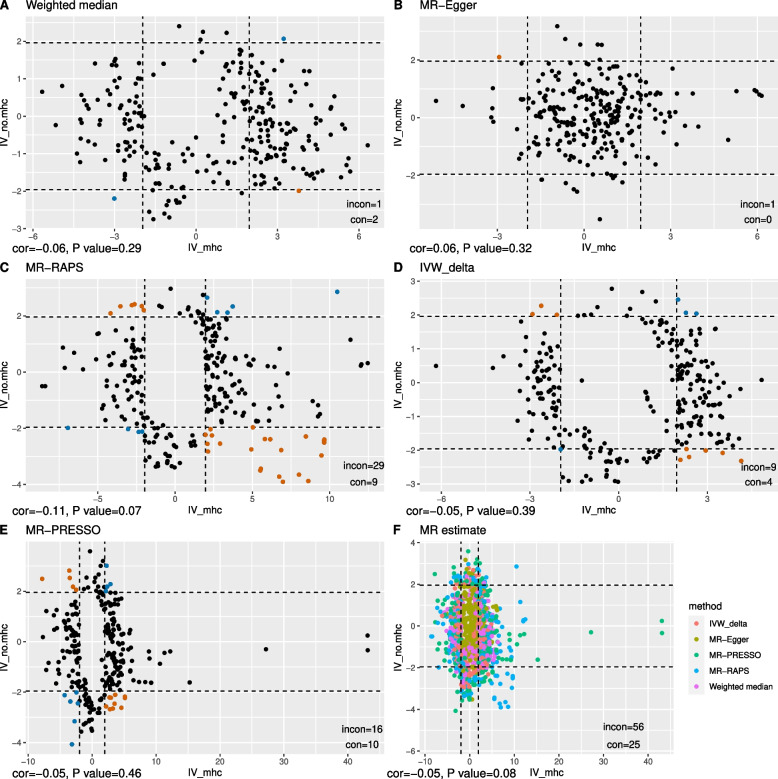
Comparisons between MR estimates inferred from IVs inside and outside the MHC region. Scatter plot of the *Z* statistics of MR estimates of traits showing suggestive causal evidence with anti-VZV IgG levels using IVs inside (IV_mhc_, horizontal axis) or outside the MHC region (IV_no.__mhc_, vertical axis). **A**–**E** The results inferred from one MR method, with blue and orange dots indicating consistent (in the same causal directions) or inconsistent (in the opposite causal directions) MR results separately. **F** The combined MR results inferred from different methods, which are suggested by different colours. The dashed lines are the 95% confidence interval (CI) of *Z* statistics. Dots outside the CI are statistically significant (*P* < 0.05) MR results. The annotation on the bottom-left of each figure shows the Spearman correlation estimate (cor) and *P*-value of the correlation test. The legend on the bottom-right of each figure represents the number of the results that are statistically consistent (con) and statistically inconsistent (incon)

In total, 271 traits had suggestive causal relationships with anti-VZV IgG (Fig. 3, Additional file 2: Table S3). Increased risks of senile cataract and disease of lens were attributed to a high level of anti-VZV IgG, using any one of the three sets of IVs. Besides, suggestive causal evidence was shown in 29 traits whether using IV_full_ or IV_no.mhc_ (Additional file 3: Figure S1), and in 32 traits whether using IV_full_ or IV_mhc_ (Additional file 3: Figure S2). Interestingly, the estimated causal effects of anti-VZV on ten traits from IV_mhc_ and from IV_no.mhc_ were in the opposite direction (Fig. 4). Overall, there was a correlation between MR estimates from IV_full_ and those from IV_no.mhc_ (*r* = 0.65, *P* = 3.7 × 10^−165^) or between those from IV_full_ and from IV_mhc_ (*r* = 0.61, *P* = 5.9 × 10^−139^). The causal associations of 6 traits estimated from MR-RAPs were in the opposite directions between IV_full_ and IV_no.mhc_ (Fig. 5C) while 143 MR estimates between IV_full_ and IV_no.mhc_ were in the same directions (Fig. 5F). The concordance of the MR estimates between IV_full_ and IV_mhc_ was pronounced to a high degree, as all the 219 MR estimates had the same signs (Fig. 6), but less so between IV_mhc_ and IV_no.mhc_ with 56 estimated causal effects in the opposite directions and no statistically significant correlations (*r* =  − 0.05, *P* = 0.08) (Fig. 7).

## Discussion

Among the traits that showed a positive causal association with anti-VZV IgG, six (Dupuytren disease, mononeuropathies of upper limb, sarcoidosis, coeliac disease, teeth problems and led to earlier onset of allergic rhinitis) were reported in the literature. In particular, the onset of herpes leading to tooth exfoliation was reported in many patients, as the virus could spread to the trigeminal nerve and cause damage to the tooth nerve [27, 28]. When the anti-VZV level was high, the increased risks of upper limp mononeuropathy could be explained by an uncommon complication of herpes zoster infections, zoster-associated mononeuropathy (ZAM) [29]. Although uncommon, herpes zoster directly leading to mononeuropathy was found in a few clinical cases. Patients having ZAM suffered from viral spreading to the peripheral nerves [30]. Dupuytren disease is a condition that affects the fascia and causes deformity to the hand, though the causes of itself remain unknown [31]. A positive association between the risk of herpes zoster and plantar fascial fibromatosis incidence was identified in the literature [32], but no causal evidence was ever reported. Coeliac disease, sarcoidosis and allergic rhinitis are the consequences of dysregulation of the immune system. Coeliac disease and sarcoidosis are caused by the immune system attacking the intestine and multiple systems, respectively. Allergic rhinitis often refers to the overreacts of the immune system in the upper respiratory tract. There has been extensive discussion about the correlation between the incidence of herpes zoster reactivations and autoimmune diseases [33, 34]. VZV reactivation can induce different types of sarcoidosis, such as scar sarcoidosis [35] and central nervous system sarcoidosis [36]. The incidence of herpes zoster was positively correlated with coeliac disease [37] and allergic rhinitis [38]. All the evidence from these studies supported our causal findings. However, the relationships between herpes infections and these autoimmune diseases might not be simply unidirectional. For example, the virus itself might increase the risk of flares in autoimmune diseases like systemic lupus erythematosus [39]. In addition, herpes zosters are more likely to reactivate in these patients as they might take immunosuppressive medications [40]. However, the immunosuppressive treatment also decreases the antibody levels in one’s body [41]. Therefore, interactions between immune responses to infections, autoimmune diseases and immunosuppressive treatments could be potentially considered in future studies.

Causal associations between VZV infections and eight traits (number of incorrect matches in pairs matching [42, 43], guilty/tense feeling [8, 44, 45], BMI [46, 47] and body/trunk fat mass and percentage) were inconclusive from previous studies. The direct causal relationships of four traits (carpal tunnel syndrome, FEV1, PEF, other specific joint disorders) and VZV were not reported before, but they may have plausible biological links [48–51]. In addition, we discovered that inguinal hernia and diabetic eye disorder may be causally associated with VZV immune response. The definitions of other serious medical conditions/disabilities diagnosed by a doctor and long-standing illness, disability or infirmity were unclear and require further explorations. Our MR results provided new insight into the causal relationships between VZV immune response and these traits, which however need to be replicated in other independent studies.

The role of the MHC region plays in MR studies had largely been overlooked [52]. In some studies, IVs in the MHC region were simply removed to minimize the risk of bias due to the pleiotropy introduced by these SNPs [15, 53]. However, some MHC SNPs may be strongly associated with exposure, especially for immunity-related traits [13]. Here, we explored the impact of MHC instruments by performing three separate MR analyses using IV_full_, IV_mhc_ and IV_no.mhc_. For the traits showing a suggestive causal relationship with anti-VZV IgG levels, there were substantial differences in the results among the three IV sets. Evidence for a correlation of the estimated causal effects was shown between IV_full_ and IV_no.mhc_ and also between IV_full_ and IV_mhc_, with approximately one-third of the traits showing statistically significant (*P* < 0.05) causal relationships with anti-VZV IgG and in the same directions. However, using the two subsets (IV_no.mhc_ and IV_mhc_) cannot fully identify all the causal relationships found using IV_full_. In fact, each subset had its specific causal findings. The number of traits that showed suggestive causal evidence with VZV immune response was almost four times larger using IVs from the MHC region than from non-MHC regions. As the IVs in the MHC regions have stronger associations with anti-VZV IgG, it was not unexpected that causal relationships were detected in a higher number of traits when using IV_mhc_. Interestingly, there was a discrepancy in causal estimates from IV_mhc_ and IV_no.mhc_ with opposite causal directions shown in ten traits. This might indicate that IVs in and outside the MHC region drive different mechanisms or intermediaries behind anti-VZV IgG levels. For example, MHC SNPs might be more involved in the immune responses in one’s body [54] while non-MHC SNPs be more associated with general health conditions that also affect the antibody levels [55]. Taken together, MHC instruments might play a different but important role in affecting the antibody levels and subsequently the clinical outcomes. Partitioning the IVs by MHC region might lead to conclusions that are different from the MR results using all the IVs. It would be advisable to investigate this separately in future MR studies. Moreover, as MHC IVs can potentially have an impact on MR analysis, imputing the region using specialist software would enhance accuracy and resolution for causal findings [56].

It is worth noting that suggestive causal relationships between some traits and anti-VZV IgG shown in this study were already reported previously (e.g. stroke [57] and systemic lupus erythematosus [39]) (Additional file 2: Table S3). However, the evidence we found was not strong enough possibly due to the complicated causal mechanisms that weakened the marginal causal effect size and therefore reduced the power to detect them in a phenome-wide MR study. Previous GWAS identified five SNPs associated with anti-VZV IgG levels [13], four (rs13197633, rs13204572, rs1048381 and rs56401801) of which were also found associated with anti-VZV IgG levels in our study. Rs34073492, also identified in previous GWAS, was however removed from analysis post-QC (MAF > 0.02).

The anti-VZV IgG levels in our study population were not likely driven by vaccination. This is because the age group enrolled in the UK national programme for shingles vaccination is between 70 and 79 years [58], while the participants for the UK biobank cohort were aged 40–69 when first enrolled.

Multiple testing decreases type I error but increases type II error [59]. It would be excessively conservative to use a stringent statistical significance threshold in MR studies which are in general of low power [60], with a risk of not detecting potentially important causal relationships. Therefore, in this exploratory study, we also reported MR results with a *P*-value less than 0.05 in the supplementary tables to minimize the reporting bias and to enable researchers to take forward our findings for further investigations in their studies.

### Limitations

We used the anti-VZV IgG level to represent both seropositivity of VZV and the lifetime exposure to the virus. The anti-VZV IgG level was expected to represent the level of VZV reactivations or immune responses to VZV, which could, however, be affected by vaccination and other factors. In our study population, the assumption of anti-VZV IgG not driven by vaccination was plausible because the age group enrolled in the UK national programme for shingles vaccination was later than those first enrolled in the UK biobank. However, the fact that anti-VZV IgG levels can be affected by other factors in addition to vaccination makes the interpretation of the measurement difficult. Except for blocking infections, antibodies may have other meaningful functions to the host. They might work interactively with cells of the immune system for a more effective protest against pathogens or contribute to immunity with different unknown mechanisms [61, 62]. A high anti-VZV IgG level may also indicate a healthy immune function. Distinct causal effects could be inferred from IV clusters when the risk is not a single entity but a composite factor with multiple components [55]. In our study, the differences in causal results inferred from IV_mhc_ and IV_no.mhc_ may be explained by different causal pathways behind VZV immunity.

All consistent causal findings (FDR < 0.05 in at least three of the five MR methods and FDR < 0.05 in both the MR methods in sensitivity analysis) were detected using IV_mhc_ but not from IVs outside the MHC region. One possible reason would be that the instruments in the MHC region had stronger associations with anti-VZV IgG. It might also be that the results were biassed due to pleiotropy.

## Conclusions

Our phenome-wide MR study found anti-VZV IgG to be a causal risk factor of 22 traits, which helped gain new insights into the causal roles of VZV-specific immunity in diseases. While some of the causal relationships have priori biological explanations, others require further investigations. When selecting IVs for MR analysis, separating SNPs from the MHC region may potentially provide information on different causal pathways, and help better understand the specific role of the MHC region in future MR studies.

## Supplementary Information

**Additional file 1: Text S1.** Traits inclusion and exclusion criteria for the phenome-wide Mendelian randomization study. **Code S1.** R code for the Mendelian Randomization analysis.

**Additional file 2: Table S1.** SNPs associated with anti-VZV IgG levels identified by the Genome-wide association scan. **Table S2.** Full results of the phenome-wide Mendelian randomization analyses of 1,370 traits. **Table S3.** Traits showed a suggestive causal relationship with anti-VZV IgG levels.

**Additional file 3: Figure S1.** Traits suggestively causally affected by anti-VZV IgG levels using IVs outside MHC region or all regions. Listed are traits with suggestive statistical evidence (*P* < 0.05) in at least three Mendelian randomization (MR) methods, using instrumental variables from all regions (IVfull) (left panel) or outside MHC region (IVno.mhc) (right panel). Odds ratio (Panel A, horizontal axis), for a binary case/control trait, represents the estimated odds ratio when the anti-VZV IgG levels increase by 9 times. Beta value (Panel B, horizontal axis), for a continuous trait, represents the estimated change in the tested trait when the anti-VZV IgG levels increase by 9 times. **Figure S2.** Traits suggestively causally affected by anti-VZV IgG levels using IVs inside MHC region or all regions. Listed are traits with suggestive statistical evidence (*P* < 0.05) in at least three Mendelian randomization (MR) methods, using instrumental variables from all regions (IVfull) (left panel) or inside MHC region (IVmhc) (right panel). Odds ratio (Panel A, horizontal axis), for a binary case/control trait, represents the estimated odds ratio when the anti-VZV IgG levels increase by 9 times. Beta value (Panel B, horizontal axis), for a continuous trait, represents the estimated change in the tested trait when the anti-VZV IgG levels increase by 9 times.

## Data Availability

Publicly available data from the UK Biobank study was analysed in this study. The datasets are available to researchers through an open application via https://www.ukbiobank.ac.uk/enable-your-research/register. Publicly available GWAS summary statistics used in this study is available at http://www.nealelab.is/uk-biobank. The R code used for Mendelian randomization analyses is in Additional file 1: Code S1.

## Abbreviations

BMI: Body mass index
FDR: False discovery rate
FEV1: Forced expiratory volume in 1-s
GWAS: Genome-wide association study
IgG: Immunoglobulin G
IV: Instrumental variable
IVW: Inverse-variance weighted
MAF: Minor allele frequency
MHC: Histocompatibility complex
MR: Mendelian randomization
MR-PheWAS: Mendelian randomization phenome-wide association study
PEF: Peak expiratory flow
SNP: Single nucleotide polymorphism
VZV: Varicella-zoster virus
ZAM: Zoster-associated mononeuropathy
