# Supplementary material for: Exploring the causal role of the immune response to varicella-zoster virus on multiple traits: a phenome-wide Mendelian randomization study
Source: BMC Med. 2023 Apr 12;21:143. doi: 10.1186/s12916-023-02843-5 (PMC10099693; doi:10.1186/s12916-023-02843-5)
Supplement: Supplementary file 1 — Additional file 1: Text S1. Traits inclusion and exclusion criteria for the phenome-wide Mendelian randomization study. Code S1. R code for the Mendelian Randomization analysis. [file 12916_2023_2843_MOESM1_ESM.docx]

**Additional File 1**

Table of Contents

[Text S1. Traits including and excluding criteria for the phenome-wide Mendelian randomization study 2](#_Toc129937330)

[Code S1. R code for the Mendelian Randomization analysis 3](#_Toc129937331)

# Text S1. Traits including and excluding criteria for the phenome-wide Mendelian randomization study

1. We include disease codes from PHESANT, FinnGen and ICD10 sources. Certain diseases such as injuries, chromosomal abnormalities and gender-determined diseases are excluded. For example, the following codes are excluded from ICD-10 sources:

O00-O9A Pregnancy, childbirth and the puerperium

P00-P96 Certain conditions originating in the perinatal period

Q00-Q99 Congenital malformations, deformations and chromosomal abnormalities

S00-T88 Injury, poisoning and certain other consequences of external causes

V00-Y99 External causes of morbidity

Z00-Z99 Factors influencing health status and contact with health services

The similar traits coded from FinnGen and PHESANT were also removed.

1. biomarkers, symptoms, signs

We also included the biomarker-related traits, such as symptoms (e.g., knee pain) and body function measurements (e.g., heel density, FEV1). The transient function measurements (e.g., lab-test, Microalbumin in urine, blood cell count, creatinine (quantile)) which cannot represent the long-term health were removed.

2.1 signs (e.g., snoring, knee pain)

2.2 symptoms (e.g., weight change during anxiety/ depression)

2.3 biomarkers

2.4 function measurement (e.g., FEV1)

2.5 disease diagnosed age (e.g., age hypertension diagnosed at)

2.6 exposure (e.g., smoked/drink, only alcohol/tobacco dependency were included)

2.7 physical measurement (e.g., heel bone mineral density, BMI)

1. All other irrelevant traits were removed from the downstream analysis. The full trait list that was included in the study can be found in the Additional file2, TableS2.

3.1 food (e.g., milk consumption, coffee intake)

3.2 activity (e.g., exercise)

3.3 environment/social status（e.g., home location/pollution, employment）

3.4 experience (e.g., year lived in a region)

3.5 irrelevant signs, measurement (e.g., hair colour, skin colour, arm length)

3.6 treatment (e.g., medication, surgeries)

3.7 time to complete (time to complete the test)

3.8 illness of others (e.g., Alzheimer's of mother)

3.9 reasons (e.g., reasons for doing things)

#

# Code S1. R code for the Mendelian Randomization analysis

library(MRPRESSO)

library(TwoSampleMR)

library(dplyr)

library(plyr)

library(this.path)

library(MendelianRandomization)

library(mr.raps)

library(stringr)

## The `mr_presso2` function is adapted from `mr_presso` function ('MRPRESSO' package) by Xinzhu Yu.

## The new function will provide the SNP rs number used for analysis as 'my_nsnp' in the output.

mr_presso2 <- function(BetaOutcome, BetaExposure, SdOutcome, SdExposure, data, OUTLIERtest = FALSE, DISTORTIONtest = FALSE, SignifThreshold = 0.05, NbDistribution = 1000, seed = NULL){

if(!is.null(seed))

set.seed(seed)

if(SignifThreshold > 1)

stop("The significance threshold cannot be greater than 1")

if(length(BetaExposure) != length(SdExposure))

stop("BetaExposure and SdExposure must have the same number of elements")

if(class(data)[1] != "data.frame")

stop("data must be an object of class data.frame, try to rerun MR-PRESSO by conversing data to a data.frame \'data = as.data.frame(data)\'")

# Functions

"%^%" <- function(x, n) with(eigen(x), vectors %*% (values^n * t(vectors)))

getRSS_LOO <- function(BetaOutcome, BetaExposure, data, returnIV){

dataW <- data[, c(BetaOutcome, BetaExposure)] * sqrt(data[, "Weights"])

X <- as.matrix(dataW[ , BetaExposure])

Y <- as.matrix(dataW[ , BetaOutcome])

CausalEstimate_LOO <- sapply(1:nrow(dataW), function(i) {

(t(X[-i, ]) %*% X[-i, ])%^%(-1) %*% t(X[-i, ]) %*% Y[-i, ]

})

if(length(BetaExposure) == 1)

RSS <- sum((Y - CausalEstimate_LOO * X)^2, na.rm = TRUE)

else

RSS <- sum((Y - rowSums(t(CausalEstimate_LOO) * X))^2, na.rm = TRUE)

if(returnIV)

RSS <- list(RSS, CausalEstimate_LOO)

return(RSS)

}

getRandomData <- function(BetaOutcome, BetaExposure, SdOutcome, SdExposure, data){

mod_IVW <- lapply(1:nrow(data), function(i) lm(as.formula(paste0(BetaOutcome, " ~ -1 + ", BetaExposure)), weights = Weights, data = data[-i, ]))

dataRandom <- cbind(eval(parse(text = paste0("cbind(", paste0("rnorm(nrow(data), data[, \'", BetaExposure, "\'], data[ ,\'", SdExposure, "\'])", collapse = ", "), ", sapply(1:nrow(data), function(i) rnorm(1, predict(mod_IVW[[i]], newdata = data[i, ]), data[i ,\'", SdOutcome,"\'])))"))), data$Weights)

colnames(dataRandom) <- c(BetaExposure, BetaOutcome, "Weights")

return(dataRandom)

}

# 0- Transforming the data + checking number of observations

data <- data[, c(BetaOutcome, BetaExposure, SdOutcome, SdExposure)]

data <- data[rowSums(is.na(data)) == 0, ]

data[, c(BetaOutcome, BetaExposure)] <- data[, c(BetaOutcome, BetaExposure)] * sign(data[, BetaExposure[1]])

data$Weights <- data$Weights <- 1/data[, SdOutcome]^2

if(nrow(data) <= length(BetaExposure) + 2)

stop("Not enough intrumental variables")

if(nrow(data) >= NbDistribution)

stop("Not enough elements to compute empirical P-values, increase NbDistribution")

# 1- Computing the observed residual sum of squares (RSS)

RSSobs <- getRSS_LOO(BetaOutcome = BetaOutcome, BetaExposure = BetaExposure, data = data, returnIV = OUTLIERtest)

# 2- Computing the distribtion of expected residual sum of squares (RSS)

randomData <- replicate(NbDistribution, getRandomData(BetaOutcome = BetaOutcome, BetaExposure = BetaExposure, SdOutcome = SdOutcome, SdExposure = SdExposure, data = data), simplify = FALSE)

RSSexp <- sapply(randomData, getRSS_LOO, BetaOutcome = BetaOutcome, BetaExposure = BetaExposure, returnIV = OUTLIERtest)

if(OUTLIERtest)

GlobalTest <- list(RSSobs = RSSobs[[1]], Pvalue = sum(RSSexp[1, ] > RSSobs[[1]])/NbDistribution)

else

GlobalTest <- list(RSSobs = RSSobs[[1]], Pvalue = sum(RSSexp > RSSobs[[1]])/NbDistribution)

# 3- Computing the single IV outlier test

if(GlobalTest$Pvalue < SignifThreshold & OUTLIERtest){

OutlierTest <- do.call("rbind", lapply(1:nrow(data), function(SNV){

randomSNP <- do.call("rbind", lapply(randomData, function(mat) mat[SNV, ]))

if(length(BetaExposure) == 1){

Dif <- data[SNV, BetaOutcome] - data[SNV, BetaExposure] * RSSobs[[2]][SNV]

Exp <- randomSNP[, BetaOutcome] - randomSNP[, BetaExposure] * RSSobs[[2]][SNV]

} else {

Dif <- data[SNV, BetaOutcome] - sum(data[SNV, BetaExposure] * RSSobs[[2]][, SNV])

Exp <- randomSNP[, BetaOutcome] - rowSums(randomSNP[, BetaExposure] * RSSobs[[2]][, SNV])

}

pval <- sum(Exp^2 > Dif^2)/length(randomData)

pval <- cbind.data.frame(RSSobs = Dif^2, Pvalue = pval)

return(pval)

}))

row.names(OutlierTest) <- row.names(data)

OutlierTest$Pvalue <- apply(cbind(OutlierTest$Pvalue*nrow(data), 1), 1, min) # Bonferroni correction

} else{

OUTLIERtest <- FALSE

my_outlier <- NULL

tt_snp <- NULL

}

# 4- Computing the test of the distortion of the causal estimate

mod_all <- lm(as.formula(paste0(BetaOutcome, " ~ -1 + ", paste(BetaExposure, collapse = "+"))), weights = Weights, data = data)

if(DISTORTIONtest & OUTLIERtest){

getRandomBias <- function(BetaOutcome, BetaExposure, SdOutcome, SdExposure, data, refOutlier){

indices <- c(refOutlier, replicate(nrow(data)-length(refOutlier), sample(setdiff(1:nrow(data), refOutlier))[1]))

mod_random <- lm(as.formula(paste0(BetaOutcome, " ~ -1 + ", paste(BetaExposure, collapse = "+"))), weights = Weights, data = data[indices[1:(length(indices) - length(refOutlier))], ])

return(mod_random$coefficients[BetaExposure])

}

refOutlier <- which(OutlierTest$Pvalue <= SignifThreshold)

if(length(refOutlier) > 0){

if(length(refOutlier) < nrow(data)){

BiasExp <- replicate(NbDistribution, getRandomBias(BetaOutcome = BetaOutcome, BetaExposure = BetaExposure, data = data, refOutlier = refOutlier), simplify = FALSE)

BiasExp <- do.call("rbind", BiasExp)

mod_noOutliers <- lm(as.formula(paste0(BetaOutcome, " ~ -1 + ", BetaExposure)), weights = Weights, data = data[-refOutlier, ])

BiasObs <- (mod_all$coefficients[BetaExposure] - mod_noOutliers$coefficients[BetaExposure]) / abs(mod_noOutliers$coefficients[BetaExposure])

BiasExp <- (mod_all$coefficients[BetaExposure] - BiasExp) / abs(BiasExp)

BiasTest <- list(`Outliers Indices` = refOutlier, `Distortion Coefficient` = 100*BiasObs, Pvalue = sum(abs(BiasExp) > abs(BiasObs))/NbDistribution)

tt_snp = data[-refOutlier, ]

} else {

BiasTest <- list(`Outliers Indices` = "All SNPs considered as outliers", `Distortion Coefficient` = NA, Pvalue = NA)

tt_snp <- NULL

}

} else{

BiasTest <- list(`Outliers Indices` = "No significant outliers", `Distortion Coefficient` = NA, Pvalue = NA)

tt_snp <- NULL

}

}

if(is.null(tt_snp) ){

nsnp <- length(data$beta.exposure)

}

else {

nsnp <- length(tt_snp$beta.exposure)

}

# 5- Formatting the results

GlobalTest$Pvalue <- ifelse(GlobalTest$Pvalue == 0, paste0("<", 1/NbDistribution), GlobalTest$Pvalue)

if(OUTLIERtest){

OutlierTest$Pvalue <- replace(OutlierTest$Pvalue, OutlierTest$Pvalue == 0, paste0("<", nrow(data)/NbDistribution))

if(DISTORTIONtest){

BiasTest$Pvalue <- ifelse(BiasTest$Pvalue == 0, paste0("<", 1/NbDistribution), BiasTest$Pvalue)

res <- list(`Global Test` = GlobalTest, `Outlier Test` = OutlierTest, `Distortion Test` = BiasTest)

} else {

res <- list(`Global Test` = GlobalTest, `Outlier Test` = OutlierTest)

}

if(nrow(data)/NbDistribution > SignifThreshold)

warning(paste0("Outlier test unstable. The significance threshold of ", SignifThreshold, " for the outlier test is not achievable with only ", NbDistribution, " to compute the null distribution. The current precision is <", nrow(data)/NbDistribution, ". Increase NbDistribution."))

} else {

res <- list(`Global Test` = GlobalTest)

}

OriginalMR <- cbind.data.frame(BetaExposure, "Raw", summary(mod_all)$coefficients)

colnames(OriginalMR) <- c("Exposure", "MR Analysis", "Causal Estimate", "Sd", "T-stat", "P-value")

if(exists("mod_noOutliers")){

OutlierCorrectedMR <- cbind.data.frame(BetaExposure, "Outlier-corrected", summary(mod_noOutliers)$coefficients)

} else{

OutlierCorrectedMR <- cbind.data.frame(BetaExposure, "Outlier-corrected", t(rep(NA, 4)))

}

colnames(OutlierCorrectedMR) <- colnames(OriginalMR)

MR <- rbind.data.frame(OriginalMR, OutlierCorrectedMR)

row.names(MR) <- NULL

my_nsnp <-cbind.data.frame(nsnp)

res <- list(`Main MR results` = MR, `MR-PRESSO results` = res,`my_nsnp` = my_nsnp)

return(res)

}

## Our VZV phenome-wide Mendelian randomization analyses

## read in exposure dataset; columns in 'VZV_format.csv': {SNP (SNP ID, variant identifier), position (position of the variant), effect_allele, beta (estimated effect size of snp-exposure association), se(estimated standard error of beta), eaf(allele frequency of the effect allele), Phenotype}

VZV_format <- read.csv("re_VZV_format.csv") ##IV_full

VZV_format_noMHC <- read.csv("re_VZV_format_noMHC.csv") ##IV_no.mhc

VZV_format_MHC <- read.csv("re_VZV_format_MHC.csv") ##IV_no.mhc

VZV_format_p8 <- read.csv("re_VZV_format_p8.csv") ##IVs with P value of association less than 5*10-8

## set the directory path to the file path containing multiple outcome datasets

file.path = paste0(this.dir(),"/","outcome_dat")

a = list.files(file.path)

dir = paste(file.path,a,sep = "/")

n = length(dir)

## read in the snp-variant map for outcome dataset; the snp-variant map was the map file matching variant name in outcome dataset to snp ID, downloaded from 'http://www.nealelab.is/uk-biobank' columns in 'snp_variant_map.csv': {SNP (SNP ID, variant identifier), variant(Variant identifier in the form "chr:pos:ref:alt", where "ref" is aligned to the forward strand.)}

snp_map <- read.csv("snp_variant_map.csv")

colnames(snp_map) <- c("SNP","variant")

## The sample size of the exposure GWAS is 6425

exposure_dat <- format_data(VZV_format)

exposure_dat$samplesize.exposure <- 6425

exposure_dat2 <- format_data(VZV_format_noMHC)

exposure_dat2$samplesize.expsoure <- 6425

exposure_dat3 <- format_data(VZV_format_MHC)

exposure_dat3$samplesize.exposure <- 6425

exposure_dat4 <- format_data(VZV_p8)

exposure_dat4$samplesize.exposure <- 6425

MRresult_VZV <- list()

MRresult_VZV_noMHC <- list()

MRresult_VZV_MHC <- list()

MRresult_VZV_p8 <- list()

## The function `mr_phewas` is created by Xinzhu Yu

## The required inputs are {IV: input formats are: ‘no.mhc’, ‘mhc’,’full’. Other inputs will be considered as sensitivity analysis; exposure dataset; snp_map file (details see above)}

## The function will generate MR results of five methods {MR-IVW,MR weighted median method, MR-Egger,MR-PRESSO,MR-RAPS} in list format

mr_phewas <- function(IV, exposure_dat, snp_map){

##analysis loop for multiple traits

for (i in 1:n){

try(outcome_dat <- read.delim(file = dir[i],header = F),silent = F)

try(if(typeof(outcome_dat$V4) == "double"){

outcome_dat <- outcome_dat[,c(1:3,5:12)]

})

try(colnames(outcome_dat) <- c("variant","minor_allele","eaf","LCV","samplesize","AC","ytx","beta","se","tstat","pval"))

outcome_dat$effect_allele <- str_sub(outcome_dat$variant, - 1, - 1)

##manually flipped beta coefficient to minor allele

outcome_dat[outcome_dat$minor_allele != outcome_dat$effect_allele,]$beta <- -1*outcome_dat[outcome_dat$minor_allele != outcome_dat$effect_allele,]$beta

outcome_dat <- subset(outcome_dat,select= -(effect_allele))

outcome_dat$effect_allele <- outcome_dat$minor_allele

try(outcome_dat <- left_join(outcome_dat,snp_map,by = "variant"))

try(outcome_dat <- format_data(outcome_dat,type = "outcome"))

datai <- steiger_filtering(datai)

datai <- datai[!datai$steiger_dir =="FALSE" & datai$steiger_pval <= 0.05,] ##Steiger filter out the SNPs with outcome effect statistically larger than the exposure

if (IV == 'full'){

datai <- harmonise_data(exposure_dat , outcome_dat,action = 1) ##action=1,assume all strands the same, because they were manually checked by the author

} else if (IV == "mhc"){

datai <- harmonise_data(exposure_dat2 , outcome_dat,action = 1)

} else if (IV == "no.mhc"){

datai <- harmonise_data(exposure_dat3 , outcome_dat,action = 1)

} else {

datai <- harmonise_data(exposure_dat4 , outcome_dat,action = 1)

}

if(length(datai$SNP) <1){

resi <- data.frame(

id.exposure= datai$id.exposure[1],

id.outcome = datai$id.outcome[1],

outcome = datai$outcome[1],

exposure =datai$exposure[1],

nsnp = "No SNPs remains after steiger",

method ="none",

b = "NA",

se = "NA",

pval = "NA",

trait = a[i],

Q = "NA",

Q_pval = "NA")

result_all <- resi

} else if(length(datai$SNP) <3){

out <- mr.raps::mr.raps.mle(b_exp = datai$beta.exposure, b_out = datai$beta.outcome, se_exp = datai$se.exposure, se_out = datai$se.outcome)

##calculate F-statistics; egger intercept

##The estimated overdispersion parameter is very small. Consider using the simple model without overdispersion.

# default parameters: over.dispersion = FALSE,#loss.function = c("l2", "huber", "tukey"),diagnostics = FALSE,pruning = TRUE,se.method = c("sandwich", "bootstrap"),k = switch(loss.function[1], l2 = NULL, huber = 1.345, tukey = 4.685),B = 1000,)

resi2 <- data.frame(

id.exposure= datai$id.exposure[1],

id.outcome = datai$id.outcome[1],

outcome = datai$outcome[1],

exposure =datai$exposure[1],

nsnp = length(datai$beta.exposure),

method ="mr.raps",

b = out$beta.hat,

se = out$beta.se,

pval = out$beta.p.value,

trait = a[i],

Q = "NA",

Q_pval = "NA")

resi <- resi2

##IVW_delta_correction loop

dati_second <- mr_input(bx = datai$beta.exposure, bxse = datai$se.exposure, by = datai$beta.outcome, byse = datai$se.outcome, exposure = datai$exposure[1], outcome = a[i], snps = datai$SNP )

IVW_res <- mr_ivw(dati_second, weights = "delta")

IVW_resi <-

data.frame(

id.exposure= NA,

id.outcome = NA,

outcome = 'outcome',

exposure = IVW_res$Exposure,

nsnp = IVW_res$SNPs,

method ="IVW_delta",

b = IVW_res$Estimate ,

se = IVW_res$StdError ,

pval = IVW_res$Pvalue,

trait = IVW_res$Outcome,

Q = IVW_res$Heter.Stat[1],

Q_pval = IVW_res$Heter.Stat[2])

resi <- rbind(resi,IVW_resi)

result_all <- resi

}else{

##heterogeneity test

hetero <- mr_heterogeneity(datai)

hetero <- hetero[,c("method","Q","Q_pval")]

##egger intercpt test

intercept <- mr_pleiotropy_test(datai)

intercept$method <- "egger_intercept"

intercept <- intercept[,c("method","pval")]

## MR weighted median; MR-egger regression

resi1 <- mr(datai,method_list = c("mr_weighted_median", "mr_egger_regression"))

try(if (nrow(resi1)!= 0){

resi1$trait <- a[i]

})

resi1 <- left_join(resi1,hetero, by ="method")

resi1 <- bind_rows(resi1,intercept)

##mr-raps

out <- mr.raps(b_exp = datai$beta.exposure,b_out = datai$beta.outcome, se_exp = datai$se.exposure,se_out = datai$se.outcome)

##make the format of results consistent with that of resi1

resi2 <- data.frame(

id.exposure= datai$id.exposure[1],

id.outcome =datai$id.outcome[1],

outcome = datai$outcome[1],

exposure =datai$exposure[1],

nsnp = length(datai$beta.exposure),

method ="mr.raps",

b = out$beta.hat,

se = out$beta.se,

pval = out$beta.p.value,

trait = a[i],

Q = "NA",

Q_pval = "NA")

resi <- rbind(resi1,resi2)

##IVW_delta_correction

dati_second <- mr_input(bx = datai$beta.exposure, bxse = datai$se.exposure, by = datai$beta.outcome, byse = datai$se.outcome, exposure = datai$exposure[1], outcome = a[i], snps = datai$SNP )

##tidy resuls format to be consitent with resi1

IVW_res <- mr_ivw(dati_second, weights = "delta")

IVW_resi <-

data.frame(

id.exposure= NA,

id.outcome = NA,

outcome = 'outcome',

exposure = IVW_res$Exposure,

nsnp = IVW_res$SNPs,

method ="IVW_delta",

b = IVW_res$Estimate ,

se = IVW_res$StdError ,

pval = IVW_res$Pvalue,

trait = IVW_res$Outcome,

Q = IVW_res$Heter.Stat[1],

Q_pval = IVW_res$Heter.Stat[2])

resi <- rbind(resi,IVW_resi)

## mr-presso

tmp <- try(presso_res <- mr_presso2(BetaOutcome = 'beta.outcome', BetaExposure = 'beta.exposure', SdOutcome = 'se.outcome', SdExposure = 'se.exposure', OUTLIERtest = TRUE, DISTORTIONtest = TRUE, data = datai, NbDistribution = 1000, SignifThreshold = 0.05),silent = T)

if(class(tmp) == "try-error"){

try(presso_resi <- data.frame(

id.exposure= NA,

id.outcome = NA,

outcome = 'outcome',

exposure = NA,

nsnp = NA,

method = 'Presso_failed',

b = NA,

se = NA,

pval = NA,

trait = a[i],

Q = NA,

Q_pval = NA))

}else{

presso_res <- mr_presso2(BetaOutcome = 'beta.outcome', BetaExposure = 'beta.exposure', SdOutcome = 'se.outcome', SdExposure = 'se.exposure', OUTLIERtest = TRUE, DISTORTIONtest = TRUE, data = datai, NbDistribution = 1000, SignifThreshold = 0.05)

tt <- presso_res[1]

tt2 <- do.call(rbind.data.frame,tt)

tt2$Exposure <- datai$exposure[1]

tt2$Outcome <- a[i]

tt3 <- do.call(cbind.data.frame,presso_res[3])

##tidy resuls format to be consitent with resi1

try(presso_resi <- data.frame(

id.exposure = NA,

id.outcome = NA,

outcome = rep('outcome',2),

exposure = tt2$Exposure,

nsnp = tt3$nsnp,

method = tt2$`MR Analysis`,

b = tt2$`Causal Estimate` ,

se = tt2$Sd,

pval = tt2$`P-value`,

trait = tt2$Outcome,

Q = NA,

Q_pval = NA))

}

try(result_all <- rbind.fill(resi,presso_resi))

}

if (IV == 'full'){

try(MRresult_VZV[[i]] <- result_all,silent = T)

return(MRresult_VZV)

} else if (IV == "mhc"){

try(MRresult_VZV_MHC[[i]] <- result_all,silent = T)

return(MRresult_VZV_MHC)

} else if (IV == "no.mhc"){

try(MRresult_VZV_noMHC[[i]] <- result_all,silent = T)

return(MRresult_VZV_noMHC)

} else {

try(MRresult_VZV_p8[[i]] <- result_all,silent = T)

return(MRresult_VZV_p8)

}

}

MRresult_VZV <- mr_phewas(IV = ’full’,exposure_dat, snp_map)

MRresult_VZV_noMHC <- mr_phewas(IV = ’no.mhc’, exposure_dat2, snp_map)

MRresult_VZV_MHC <- mr_phewas(IV = ’mhc’, exposure_dat3, snp_map)

MRresult_VZV_p8 <- mr_phewas(IV = ’sensitivity’, exposure_dat4, snp_map)

MRresult_VZV <- do.call(rbind.data.frame,MRresult_VZV)

MRresult_VZV_noMHC <- do.call(rbind.data.frame,MRresult_VZV_noMHC)

MRresult_VZV_MHC <- do.call(rbind.data.frame,MRresult_VZV_MHC)

MRresult_VZV_p8 <- do.call(rbind.data.frame,MRresult_VZV_p8)

output_dir1 = paste0(this.dir(),"/","MRresult_VZV.rdata")

output_dir2 = paste0(this.dir(),"/","MRresult_VZV_noMHC.rdata")

output_dir3 = paste0(this.dir(),"/","MRresult_VZV_MHC.rdata")

output_dir4 = paste0(this.dir(),"/","MRresult_VZV_p8.rdata")

save(MRresult_VZV,file = output_dir1)

save(MRresult_VZV_noMHC,file = output_dir2)

save(MRresult_VZV_MHC,file = output_dir3)

save(MRresult_VZV_p8,file = output_dir4)
