# Supplementary material for: Exploring the causal role of the immune response to varicella-zoster virus on multiple traits: a phenome-wide Mendelian randomization study
Source: BMC Med. 2023 Apr 12;21:143. doi: 10.1186/s12916-023-02843-5 (PMC10099693; doi:10.1186/s12916-023-02843-5)
Supplement: Supplementary file 3 — Additional file 3: Figure S1. Traits suggestively causally affected by anti-VZV IgG levels using IVs outside MHC region or all regions. Listed are traits with suggestive statistical evidence (P < 0.05) in at least three Mendelian randomization (MR) methods, using instrumental variables from all regions (IVfull) (left panel) or outside MHC region (IVno.mhc) (right panel). Odds ratio (Panel A, horizontal axis), for a binary case/control trait, represents the estimated odds ratio when the anti-VZV IgG levels increase by 9 times. Beta value (Panel B, horizontal axis), for a continuous trait, represents the estimated change in the tested trait when the anti-VZV IgG levels increase by 9 times. Figure S2. Traits suggestively causally affected by anti-VZV IgG levels using IVs inside MHC region or all regions. Listed are traits with suggestive statistical evidence (P < 0.05) in at least three Mendelian randomization (MR) methods, using instrumental variables from all regions (IVfull) (left panel) or inside MHC region (IVmhc) (right panel). Odds ratio (Panel A, horizontal axis), for a binary case/control trait, represents the estimated odds ratio when the anti-VZV IgG levels increase by 9 times. Beta value (Panel B, horizontal axis), for a continuous trait, represents the estimated change in the tested trait when the anti-VZV IgG levels increase by 9 times. [file 12916_2023_2843_MOESM3_ESM.docx]

**Additional file 3**

Table of Contents

[Figure S1. Traits suggestively causally affected by anti-VZV IgG levels using IVs outside MHC region or all regions 2](#_Toc129728005)

[Figure S2. Traits suggestively causally affected by anti-VZV IgG levels using IVs inside MHC region or all regions 4](#_Toc129728006)

#

# Figure S1. Traits suggestively causally affected by anti-VZV IgG levels using IVs outside MHC region or all regions

Listed are traits with suggestive statistical evidence (*P* < 0.05) in at least three Mendelian randomization (MR) methods, using instrumental variables from all regions (IV_full_) (left panel) or outside MHC region (IV_no.mhc_) (right panel). Odds ratio (Panel A, horizontal axis), for a binary case/control trait, represents the estimated odds ratio when the anti-VZV IgG levels increase by 9 times. Beta value (Panel B, horizontal axis), for a continuous trait, represents the estimated change in the tested trait when the anti-VZV IgG levels increase by 9 times.

# Figure S2. Traits suggestively causally affected by anti-VZV IgG levels using IVs inside MHC region or all regions

Listed are traits with suggestive statistical evidence (*P* < 0.05) in at least three Mendelian randomization (MR) methods, using instrumental variables from all regions (IV_full_) (left panel) or inside MHC region (IV_mhc_) (right panel). Odds ratio (Panel A, horizontal axis), for a binary case/control trait, represents the estimated odds ratio when the anti-VZV IgG levels increase by 9 times. Beta value (Panel B, horizontal axis), for a continuous trait, represents the estimated change in the tested trait when the anti-VZV IgG levels increase by 9 times.
